# A Dietary Pattern with High Sugar Content Is Associated with Cardiometabolic Risk Factors in the Pomak Population

**DOI:** 10.3390/nu11123043

**Published:** 2019-12-13

**Authors:** Aliki-Eleni Farmaki, Nigel W Rayner, Maria Kafyra, Angela Matchan, Kyriaki Ntaoutidou, Pournar Feritoglou, Antonis Athanasiadis, Arthur Gilly, Vasiliki Mamakou, Eleni Zengini, Maria Karaleftheri, Eleftheria Zeggini, George Dedoussis

**Affiliations:** 1 Department of Nutrition and Dietetics, School of Health Science and Education, Harokopio University, 17671 Athens, Greece or mariakafira1@gmail.com (M.K.); ericadaoutd@gmail.com (K.N.); pournarferitoglou@gmail.com (P.F.); 2 MRC Unit for Lifelong Health & Ageing, Institute of Cardiovascular Science, University College London, London WC1E 7HB, UK; 3 Institute of Translational Genomics, Helmholtz Zentrum Munchen, German Research Center for Environmental Health, D-85764 Neuherberg, Germany; wrayner@well.ox.ac.uk (N.W.R.); arthur.gilly@helmholtz-muenchen.de (A.G.); eleftheria.zeggini@helmholtz-muenchen.de (E.Z.); 4 Wellcome Trust Centre for Human Genetics, University of Oxford, Roosevelt Drive, Oxford OX3 7BN, UK; 5 Oxford Centre for Diabetes, Endocrinology and Metabolism, Churchill Hospital, Headington, Oxford OX3 7LE, UK; 6 Wellcome Sanger Institute, The Morgan Building, Wellcome Trust Genome Campus, Hinxton, Cambridge CB10 1HH, UK; 7 Genomics England, Queen Mary University of London, Dawson Hall, Charterhouse Square, London EC1M 6BQ, UK; angela.matchan@genomicsengland.co.uk; 8 Echinos Medical Centre, Xanthi 67300, Greece; antonios.athanasiadis@1070.syzefxis.gov.gr (A.A.); mkaraleftheri@gmail.com (M.K.); 9 Dromokaiteio Psychiatric Hospital of Athens, Chaidari, Athens 12461, Greece; vmamakou@hotmail.com (V.M.); elenizegini1@gmail.com (E.Z.); 10 Medical School, National and Kapodistrian University of Athens, Goudi, Athens 11527, Greece; 11 Department of Human Metabolism, University of Sheffield, Sheffield S10 2TN, UK

**Keywords:** dietary patterns, cardiovascular risk, isolated population, Pomak, HELIC

## Abstract

The present study describes the geographically isolated Pomak population and its particular dietary patterns in relationship to cardiovascular risk factors. We collected a population-based cohort in a cross-sectional study, with detailed anthropometric, biochemical, clinical, and lifestyle parameter information. Dietary patterns were derived through principal component analysis based on a validated food-frequency questionnaire, administered to 1702 adult inhabitants of the Pomak villages on the Rhodope mountain range in Greece. A total of 69.9% of the participants were female with a population mean age of 44.9 years; 67% of the population were overweight or obese with a significantly different prevalence for obesity between men and women (17.5% vs. 37.5%, respectively, *p* < 0.001). Smoking was more prevalent in men (45.8% vs. 2.2%, *p* < 0.001), as 97.3% of women had never smoked. Four dietary patterns emerged as characteristic of the population, and were termed “high in sugars”, “quick choices”, “balanced”, and “homemade”. Higher adherence to the “high in sugars” dietary pattern was associated with increased glucose levels (*p* < 0.001) and increased risk of hypertension (OR (95% CI) 2.61 (1.55, 4.39), *p* < 0.001) and nominally associated with high blood glucose levels (OR (95% CI) 1.85 (1.11, 3.08), *p* = 0.018), compared to lower adherence. Overall, we characterize the dietary patterns of the Pomak population and describe associations with cardiovascular risk factors.

## 1. Introduction

The World Health Organization reports that cardiovascular disease (CVD) is the number one cause of death globally and largely caused by risk factors that can be controlled, treated, or modified, such as high blood pressure, cholesterol, obesity, tobacco use, physical inactivity, an unhealthy diet, and diabetes [1]. The inverse relationship between the consumption of a plant-based diet and CVD prevalence has previously been established from cross-sectional, prospective, case-control [2,3], and clinical studies, an effect attributed to dietary fiber decreasing serum cholesterol levels [4,5] and the antioxidant compounds that predispose to lipoprotein oxidation [5,6]. On the other hand, compounds usually encountered in meat-based diets, such as saturated and trans fatty acids, are associated with increased risk of developing atherosclerosis [7]. 

Diet is generally regarded as a major contributing factor to CVD prevention and treatment, and is considered to be the most important environmental component affecting hypertension [8]. In this context, the DASH (Dietary Approaches to Stop Hypertension) diet, characterized by higher intake of fruits, vegetables, low-fat dairy, grains, fish, and poultry, has been shown to be effective in reducing both systolic and diastolic blood pressure levels [9,10]. The study of dietary patterns represents a more holistic approach to the investigation of exposure–disease associations, as nutrients are not consumed in isolation, but rather as part of many different meals consisting of foods with a variety of both micro- and macronutrients. Principal component analysis (PCA) is a data-driven approach that reduces the large number of food variables into a smaller set that captures the major dietary traits of a population [11].

The Pomaks have, for centuries, inhabited the mountains of Thrace, an area in the northeast of modern Greece. The Pomaks live across the Rhodope mountain range and, following World War II, their populations were split between Greece and Bulgaria. In Greece, Pomaks dwell in three main areas: villages to the north of Xanthi, Komotini, and Evros [12]. They represent a Muslim minority in Greece with uncertain origins [13]. According to the most dominant theory, the Pomaks are descendants of the natives of the Rhodope Mountains [14].

Although the population has been the object of extensive research, in terms of politics and folklore culture, to date there have not been any studies on their health and lifestyle. The main objective of this work was to conduct a well-phenotyped, population-based study in order to facilitate epidemiological analyses with respect to cardiovascular disease. The present study investigates the potential associations between dietary patterns derived from analyzing the eating habits of the local Pomak populations and cardiometabolic risk factors, as well as diabetes prevalence.

## 2. Materials and Methods 

### 2.1. Study Population 

The Pomak target population comprised adults originating from villages located in the regional unit of Xanthi (population estimated to be 25,000 in total). 

**Figure 1:**
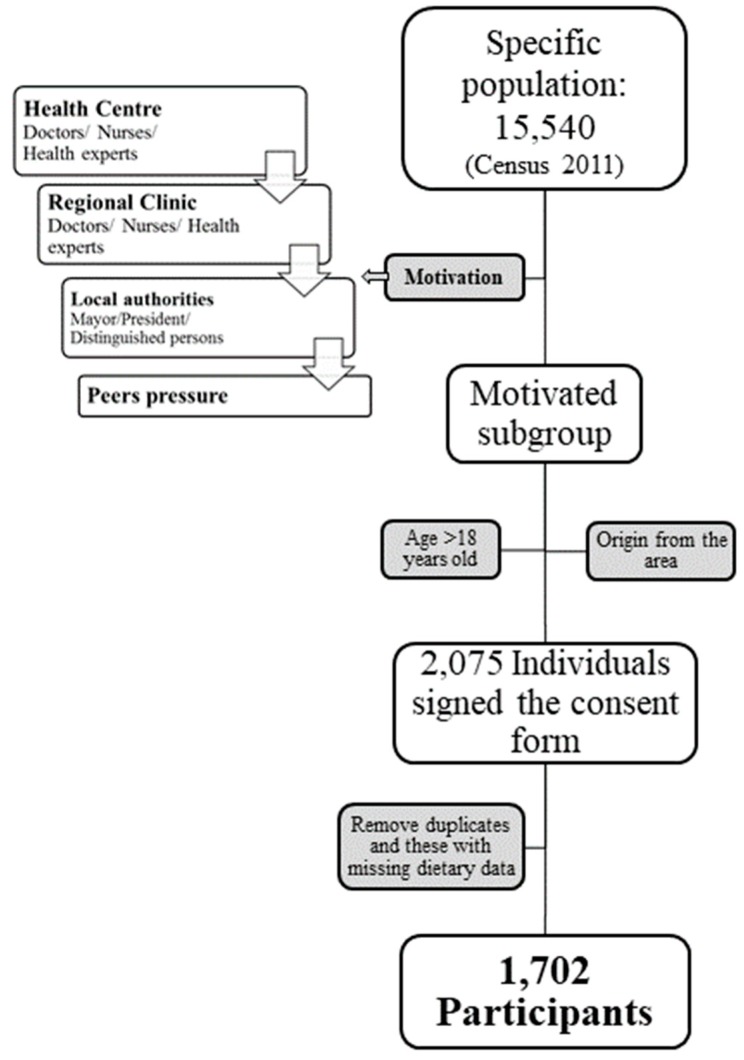
Recruitment flowchart.

We contacted the local Health Centre in Echinos and, with the cooperation of the local doctors and nurses, a total of 1702 volunteers were recruited between July 2010 and March 2013. The recruitment flowchart is summarized in Figure 1. The Bioethics Committee of Harokopio University of Athens approved the study protocol and all subjects signed a voluntary informed consent form, according to the standards of the World Medical Association’s Declaration of Helsinki for ethical principles for medical research involving human subjects. All participants were interviewed by trained personnel using a standardized questionnaire. The questionnaire included clinical assessment and anthropometric measurements, as well as questions on demographic and lifestyle characteristics, detailed self-reported medical history, physical activity, and dietary habits. The medical history and corresponding medication were cross-checked by trained clinicians.

### 2.2. Bioche Mical Analysis 

In the regional medical centers, blood samples were collected from the antecubital vein by trained health professionals following blood pressure measurement. All blood draws were conducted in the morning after an overnight fast. Serum and plasma were isolated following centrifugation at 3000 rpm for 10 min and stored at −80 °C for future analyses. Biochemical factors were assessed by Bioiatriki Healthcare Group, Athens, using enzymatic colorimetric assays, including glucose (hexokinase method), total cholesterol (cholesterol oxidase–phenol aminophenazone method), high-density lipoprotein (HDL) cholesterol, and triglycerides (glycerol-3-phosphate oxidase–phenol aminophenazone). Insulin was measured via chemiluminescence and low-density lipoprotein (LDL) cholesterol levels were calculated according to the Friedewald equation [15]. 

### 2.3. Anthropometric Measurements

The anthropometric measurements, including weight, height, waist circumference (WC), and hip circumference, were collected by trained dietitians using standardized techniques and equipment [16]. Body weight was measured using calibrated analog scales. For height and other anthropometric measurements, a stable tape was used.

Body mass index (BMI) was calculated as weight (kg) divided by height^2^ (m^2^). BMI was used to classify subjects as underweight (BMI < 18.5 kg/m^2^), normal weight (18.5 < BMI < 24.9 kg/m^2^), overweight (25.0 < BMI < 29.9 kg/m^2^) and obese (BMI > 30.0 kg/m^2^). Waist-to-hip ratio (WHR) was also calculated. 

### 2.4. Demographic and Lifestyle Information

Information on marital status (married, separated, divorced, single, or widowed) was collected. Educational level was defined as no education, primary, secondary, or tertiary education, and the total number of years in education was recorded. Information on smoking habits was also collected; subjects were classified as current smokers, ex-smokers, and those who had never smoked. Pack-year was used as the unit for measuring the amount a person smoked over a long period of time and calculated by multiplying the number of packs of cigarettes (20 cigarettes per pack) smoked per day by the number of years the person has smoked.

### 2.5. Physical Activity Assessment 

Physical activity was evaluated using the Harokopio Physical Activity Questionnaire (HAPAQ) [17]. The questionnaire collects information on self-reported physical activity over the preceding week, and examines the time spent in light, moderate, and high-intensity activities as well as time asleep. The questionnaire is based on metabolic equivalents for all activities in the preceding week, including those at work, leisure, and at rest or asleep, thus allowing the estimation of a mean physical activity level (PAL) and a mean daily energy expenditure (EE). These were used to quantify the intensity of activity and consequently to calculate energy expenditure from the activities identified in the questionnaire using the method of metabolic equivalents (MET) [18].

Based on the HAPAQ and its corresponding calculations for estimating the variables from the data, a program entitled “the Physical Activity Program” was developed to automate and accelerate the calculations to derive these estimations (available online: http://www.well.ox.ac.uk/~wrayner/tools/). The program is written in Perl and can be used to transform physical activity data into PAL, total EE, and many of the other intermediate calculations. 

### 2.6. Dietary Assessment

Dietary information was collected via a validated semiquantitative 76-item food-frequency questionnaire (FFQ) [19]. All participants reported their daily, weekly, and monthly average intake of a variety of foods during the past year. The former served as the basis for quantifying the frequency of the consumption of the food items, on the basis of servings per week and according to the dietary guidelines for adults in Greece [20]. 

The above-mentioned transformations (foods and food groups in servings) were calculated programmatically. 

Energy intake (EI) was extracted from the FFQ [21]. In order to identify and exclude energy under-reporters, the Goldberg et al. method [21,22] was used, according to the equation:

(1) EI:BMR > PAL xexp[−2  x S100n ] where  S=CVwEI2d+CVwB2+CVtP2

where $CV_{wEI}^{\;}:$ within-individual coefficient of variation for energy intake; d: the number of days of diet assessment (for FFQs, d approaches infinity and so $\frac{CV_{wEI}^{2}}{d}$ = 0); $CV_{wB}^{}:$ coefficient of variation of measured or predicted BMR; $CV_{tP}^{\;}:$ coefficient of variation for PAL; and *n*: number of individuals for which intake is examined. PAL is a multiple of BMR and therefore represents the activity level of each subject as a ratio of BMR (PAL = EE:BMR) [23]. The coefficient of variation (CV) for estimated BMR has been determined to be 8% [24] while the CV for the PAL is 12.5% [25]. Thus, the above equation for our study applies as follows:

(2) $$CV_{wEI}^{\;}=0,\;CV_{wB}^{\;}=8,\;CV_{tP}^{\;}=12.5$$

(3) EI:BMR>EE:BMR xexp[−2  x S100n ] => EI:EE>exp[−2  x S100n ], where S=(8) 2+(12.5) 2

so EI:EE > 0.74 provided that evaluation is conducted on an individual basis (*n* = 1). Individuals whose energy intake to energy expenditure (EI/EE) ratio was below this cut-off point (0.74) were defined as under-reporters and excluded from subsequent dietary analyses (*N* final = 1513).

### 2.7. Statistical Analysis

The data handling, basic processing, and descriptive characteristic analysis were carried out in R [25,26,27]. Sex differences were assessed using the Wilcoxon test. Distribution normality was checked with the Shapiro–Wilk test applying sex stratification where needed. Hypothesis testing was performed using the chi-squared test for categorical variables and Student’s T test or Mann–Whitney for continuous variables in two different groups.

Outliers were defined as values that exceeded three (3) standard deviations (SD) from the mean and were removed from subsequent analyses. Binary variables were created dividing subjects into normal and pathological levels of anthropometric (WC, WHR) [28], clinical (systolic blood pressure-SBP, diastolic blood pressure-DBP), and biochemical (total cholesterol, LDL, glucose, insulin) indices [29], taking into account the corresponding diagnosis and medication information.

Principal component analysis (PCA) was undertaken to identify underlying dietary patterns [30]. In order for PCA to be effective in assessing food patterns, strong correlations between the predictive variables should exist. The Kaiser–Meyer–Olkin (KMO) criterion was calculated at 0.76, which is close to one implying high interrelationships between food variables and therefore suitability of the data set for PCA. The orthogonal rotation (varimax option) was used to derive optimal noncorrelated components (dietary patterns). Fifteen foods and food groups were used from the entire database. The Kaiser criterion was used in order to decide on the number of components to retain, according to which, the number of components that should be retained from PCA is equal to the number of Eigen values greater than 1. In our analysis, a four-component (food pattern) solution was selected. Based on the factor loadings/correlation coefficients that represent the correlation of each predicting variable with the dietary pattern score, higher absolute values indicate that the variable contributes more to the construction of a particular pattern. Dietary patterns were named according to the scores of the predicting variables with the highest correlation to the component/pattern (>|0.4|). The median was used to divide pattern scoring into two groups: low and high. The PCA was performed using SPSS [31] and four dietary patterns emerged: the “high in sugars”, the “quick choices”, the “balanced”, and the “homemade” patterns.

Multiple linear regression was used to examine the association between cardiometabolic indices and dietary patterns. In particular, four different models were applied: Model 1 was unadjusted; Model 2 included adjustment for age, sex, and BMI; Model 3, as per Model 2, with the additional adjustment for corresponding medication (for hypertension, hyperlipidemia, and diabetes), smoking in pack-years, and EI/EE ratio; and Model 4, as per Model 3 with the additional adjustment for the rest of the dietary patterns. As these patterns are statistically independent, it is possible for an individual to have high or low scores on more than one pattern at the same time and therefore all the patterns might act in concert [32]. The results from the linear regression models are presented as beta coefficients (β) and 95% confidence intervals (CI). Moreover, participants’ dietary component scores were categorized into tertiles so that, for each dietary component, the third quartile included individuals whose dietary habits were mostly adherent to that particular pattern. Based on the statistically significant relationships provided by the linear regression models tested, logistic regression analysis was further performed to evaluate the association between the tertiles of each dietary component and the probability of having pathological levels of various cardiometabolic indices, according to the Third Report of the National Cholesterol Education Program (NCEP) [18]. The results of the logistic regression models are presented as odds ratios (OR) and 95% CI. The linear and logistic regressions were performed in SPSS [31]. In all statistical analyses, the level of nominal significance was set at *p* = 0.05. The adjusted threshold after multiple testing was set to (0.05/4 parameters examined, i.e., dietary patterns =) 0.0125. 

## 3. Results

The anthropometric and clinical characteristics of the population are presented in Table 1. Of the 1702 participants, 30.7% were male and 69.3% female. The mean age of the population was 44.9 years and male volunteers were on average 5.5 years older than women (*p* < 0.001). In addition, men were found to have greater WC (96.1 vs. 90.4 cm, *p* < 0.001) and WHR (0.94 vs. 0.84, *p* = 3.0E-80) than women. However, women showed a higher BMI compared to men (28 vs. 26.4 kg/m^2^, *p* < 0.001). A larger proportion of men were categorized as overweight (44.7% vs. 31.7%), while the proportion of obese women in the population was much greater than for men (25.9% vs. 5.5%, *p* < 0.001). The mean value for systolic blood pressure was 131.2 mmHg, with men presenting significantly higher levels than women (133.3 vs. 130.1 mmHg, *p* = 0.001). Refined cereals, dairy, vegetables, and sweets showed the greatest weekly consumption of all the food groups.

Hypertension and hyperlipidemia were the most prevalent disorders in the population (Table 2). A higher percentage of men had hyperlipidemia (19.5% vs. 15.4%, *p* = 0.036), presbyopia (11.1% vs. 6.2%, *p* < 0.001), type 2 diabetes (10.2% vs. 6.1%, *p* = 0.003), and coronary heart disease (11.7% vs. 4.6%, *p <* 0.001) compared to women, while depression (11.7% vs. 7.5%, *p* = 0.008), hypothyroidism (11.5% vs. 2.1%, *p <* 0.001), and migraine (8.8% vs. 4.8%, *p* = 0.004) were more prevalent in women.

Table 3 shows the sociodemographic and lifestyle characteristics of the Pomak population. The vast majority of the participants was married (86.7%) and reported primary education (72.2%). The proportion of illiteracy was higher in women than in men (13.2% vs. 5.8%, *p* < 0.001). The proportion of both current and former smokers was 20 and 40 times higher in men compared to women, respectively, while the vast majority of women (97.3%) and one third of men (35%) had never smoked (*p* < 0.001).

PCA highlighted four different dietary patterns (components), the factor loadings of which are presented in Table 4. Alcohol and pork consumption were excluded as their consumption in the Pomak population was insignificant due to religious reasons. Fifteen out of the 48 remaining foods or food groups were included in the PCA as highly intercorrelated. The KMO criterion was 0.72, and 52% of the total variance was explained by the four dietary components. The components were defined according to the higher absolute scores that indicate greater contribution to each component. Thus, there are: the “high in sugars” dietary pattern (component 1), which includes fresh fruits, milky sweets, chocolate, beverages, juices, and starchy sweets; the “quick choices” dietary pattern (component 2), which is composed of processed red meat, ready-made pies, refined breads, and fries; the “balanced” dietary pattern (component 3), that consists of refined pasta, white rice, vegetables, legumes, and red meat; and the “homemade” dietary pattern (component 4), that is characterized by homemade pies, full-fat cheese, and potato consumption. The median of the scores for each component was used to divide the population into two groups: low and high adherence to the particular pattern.

As presented in Table 5, women had significantly higher HDL cholesterol (*p* < 0.001) and lower triglyceride levels (*p* < 0.001) than men. The group with the higher scores in the “high in sugar” pattern and the balanced diet had elevated mean glucose levels. Mean HDL cholesterol was lower in the group with higher adherence scores to the “high in sugar” pattern, while LDL and total cholesterol levels were found to be higher in the group with lower adherence scores to the “quick choices” dietary pattern. The group with higher scores in the “high in sugar” pattern presented lower triglycerides levels compared to the group with lower scores in the same pattern.

The multiadjusted linear regression results are shown in Table 6. In particular, component 1 with the high sugar content was associated with higher SBP (*β* = 2.47, *p* < 0.001), DBP (*β* = 1.46, *p* < 0.001), and glucose levels (*β* = 3.44, *p* < 0.001). Additionally, the high in sugars diet was found to be significantly associated with increased LDL cholesterol but the association was found not significant after adjustment for the remaining components of the diet. Component 2, representing the quick choices/junk food pattern, was found to be associated with increased insulin levels, albeit without high statistical significance (*β* = 0.73, *p* = 0.025). Component 3, representing the balanced diet, was significantly associated with reduced SBP (*β* = −1.59, *p* = 0.001). Component 4, the homemade dietary pattern, was found to be significantly associated with higher HDL levels, after adjusting for confounders, but the effect was nonsignificant when the remaining dietary patterns were taken into account.

Logistic regression analyses, summarized in Table 7, showed that higher adherence to the high in sugars dietary pattern was nominally associated with increased risk of low HDL (OR [95%CI] 1.84 [1.24,2.75], *p* = 0.003) and systolic hypertension (OR [95%CI] 2.40 [1.38,4.17], *p* = 0.002) and statistically significantly associated with increased risk for diastolic hypertension (OR [95%CI] 2.61 [1.55,4.39], *p* < 0.001), compared to lower adherence (*p*-values are here compared to the adjusted cut-off of *p* = 0.0125.) 

## 4. Discussion

This is the first report of a cross-sectional cohort that describes the geographically and religiously isolated, genetically homogeneous [33] population from the Pomak villages in Xanthi, as part of the HELIC-Pomak study (www.helic.org). One advantage of isolated populations is that they are characterized by environmental homogeneity [34] and thus a smaller sample size is required to detect an association with sufficient statistical power. 

The Pomak population was found to be overweight, in keeping with observations from the general Greek population in the ATTICA study [35]. The ATTICA study is a population-based health and nutrition survey, which attempts to evaluate several cardiovascular risk factors in the greater urban area of Athens, Greece [36]. One third (31.3%) of the Pomak population was obese, while the corresponding percentage from the Greek Muslim community in the MetS-Greece study from the same area was 63.6% [37]. The discrepancy might be due to the sample size and the population origin, as the MetS study only included 300 individuals from inside and around the city of Xanthi, while in our study we included 1702 individuals from many different villages including Kentavros, Glafki, Echinos, Pachni, Dimario, Melivia, Sminthy, Thermes, Miki, Oreo, and other Pomak villages. Inhabitants of more distant villages have preserved traditional characteristics in many aspects of their lives, while the residents of more urbanized areas and cities conform more closely to the mainstream Greek lifestyle. Consumption of red meat, dairy, and sweets was 2–3 times higher in the Pomak population compared to that of the Attica general population [38], while vegetables, fruits, legumes, and potatoes intake was 2–4 times lower. 

The proportion of individuals with hypertension (26.1%) found in the present study was comparable with that of other Greek studies [36,37,38], but much lower than the ones reported (80%) by other isolated, but older, populations, such as the one on the island of Vis in Croatia [39]. Occurrence of hyperlipidemia (16.7%) was in the same direction and magnitude compared to the ATTICA study [38] which found a higher prevalence of the disease (43%). The prevalence of type 2 diabetes (7.3%) was comparable to that of the general Greek population [36] and also to the one of the aforementioned isolated population from the island of Vis, Croatia [39], but half of that reported (14.6%) in the chronic disease records of the local medical center [40]. However, this apparent discrepancy may be due to the fact that these records only included three areas/villages.

The overwhelming majority of the population was married (86%), indicating the traditional position of the family within the society, a trend which is attenuated in more urban areas [36]. Most of the participants had attained primary education, with more men being educated than women, a characteristic unlike that of the urban Greek population [41] which reports almost double the number of total years in education. The smoking habits of men were similar to those of the general population of Attica [42], while almost all women (97%) had never smoked, hinting at the preservation of a past etiquette that indicates the traditional character of this tobacco farming population. 

It is noteworthy that the traditional lifestyle of the populations inhabiting the Pomak villages has been extensively studied in various locations, usually referring to factors such as eating habits, which may differ slightly or considerably among the different villages. 

In this study, the characterization of the unique diet of the Pomak population was achieved through a posteriori technique, PCA analysis, generating patterns using empirically obtained data [43]. Thus, the derived patterns reflect the most representative eating habits of the population. 

The predominant dietary pattern is the “high in sugars” pattern (16.3% of the variance explained), and is very typical and closely connected with the sociocultural aspect of food in the Pomak population. Sweets, which are the main compound of the pattern, are offered after funerals in the form of chocolate or Turkish delight. In addition, increased sweets consumption is highlighted by the existence of a festival dedicated to the offering and the consumption of sweets, the “Seker Bayrami” or “Bayram of Sweets” [12]. Bayram is the word used for festivals or holidays and is applicable to secular and mainly religious celebrations in Islam. In the present study, this particular pattern, which was rich in sugars, was negatively associated with HDL cholesterol levels. A cross-sectional study evaluating data from the National Health and Nutrition Examination Survey 1999–2006 also reported similar findings [44]. The resulting low HDL cholesterol concentrations could be driven by the role of refined carbohydrates on the pathway that decreases insulin sensitivity, increases visceral adiposity, and stimulates hepatic de novo lipogenesis [45]. 

The former pattern was also found to be associated with increased systolic and diastolic blood pressure. According to a systematic review and meta-analysis of six prospective cohorts, sugar-sweetened beverage consumption was indeed found to be associated with a modest risk of developing hypertension [46]. A different systematic review and meta-analysis of eight studies further demonstrated statistically significant associations between sugary beverage consumption and hypertension [47]. Moreover, habitual consumption of fruit juice was found to be associated with higher central blood pressure and central pulse pressure [48], suggesting the overconsumption of added fructose as the main cause [49,50]. As expected, the “high in sugars” pattern was also associated with increased glucose levels. This is consistent with the results from the EPIC-InterAct study where sugar-sweetened soft drinks were associated with an increased prevalence of type 2 diabetes [51]. The European Prospective Investigation into Cancer and Nutrition (EPIC) study is one of the largest cohort studies in the world, with participants from 10 European countries, and is designed to investigate the relationships between dietary, environmental, and lifestyle factors, and the incidence of chronic diseases. The EPIC-Netherlands follow-up study stated that carbohydrate (hazard ratio 1.15) and starch (hazard ratio 1.25) intake was related to increased diabetes risk [52]. Bringing these together, it is apparent that dietary sugars have many unfavorable effects on a whole spectrum of metabolic disorders that take place in CVD and metabolic syndrome [53]. 

The “high in sugars” pattern observed in the Pomak population is associated with most of the components of metabolic syndrome, while in the ATTICA study, the “sweets” pattern was not associated with any aspect of metabolic syndrome [54]. One possible reason for this discrepancy could be the differencing tools used for dietary assessment (72 vs. 156 items FFQ) and the diversity of the compounds composing each particular pattern/component. The fat content of the milky sweets and chocolates in the “high in sugars” pattern of the Pomak population may intensify the negative effects on cardiovascular risk, while the specific food compounds of the “sweets” pattern of the ATTICA study are not known. 

High fat consumption is mainly represented by the “quick choices pattern”, which includes processed meat, fries, and ready-made pies, as well as refined breads, comprising a fast/junk food pattern. This particular pattern was nominally associated with increased levels of insulin, a finding consistent with current literature, suggesting meat consumption to be associated with hyperinsulinaemia and type 2 diabetes [38,55]. The total fat, mainly consisting of the saturated fat of the meat and the trans fatty acids of the fried potatoes, is believed to contribute to the increased prevalence of insulin resistance and type 2 diabetes [56,57,58]. 

The “balanced pattern” represents the consumption of rice, pasta, red meat, vegetables, and legumes. Rice is a traditional food of the Pomak population which, in the form of pilaf, is the main dish at ceremonies and celebrations like funerals and weddings. Red meat, such as lamb and beef, has had a central role in the social life of the Pomaks throughout their history, with the dedication of an entire religious festival to its consumption, called “Kurban Bayramı” or “Sacrifice Bayram” [12]. The former represents a prudent dietary pattern that incorporates customs and traditions of Pomaks and is found to be associated with reduced systolic and diastolic blood pressure. Similar results have been reported by the ATTICA study [42], where the Mediterranean diet was associated with a lower risk of hypertension (OR = 0.74, *p* = 0.021). Pala et al. found that hypertension was associated with adherence to the pasta and meat pattern [59]. The opposite findings demonstrated in the present study could be attributed to the additional constituents of the pasta and meat pattern such as other animal fats, wine, bread, processed meat, and pork. Moreover, the presence of vegetables and legumes in the Pomak “balanced pattern” may play a protective role against CVD. The “balanced pattern” was also associated with lower LDL cholesterol levels, a finding consistent with current literature [60]. 

The cross-sectional nature of this study does not allow for cause/effect implications to be drawn or generalization of the results from this particular Pomak population. Selection bias should also be considered as a limitation of our study without any data for comparison between responders and nonresponders. Another limitation is that the medical history and medication information come from self-reported data, subjected to possible under-reporting or memory bias. In addition, the FFQ used has not been validated in this specific population, although it has been validated in the general Greek population [21]. Moreover, the principal component analysis is partly underpinned by subjective decisions like how many foods and/or food groups have been included, how many components have been retained, and/or the nomenclature of the patterns, which is also a potential limitation. Seven outcomes of interest have been tested, using the stringent Bonferroni corrected *p*-value of 0.0125. Using this significance threshold, the associations of the “high in sugar” dietary pattern with SBP, DBP, and glucose levels and the association of the “balanced” dietary pattern with SBP levels, are the only ones to remain significant. The association between the “quick choices” dietary pattern and insulin becomes suggestive as it no longer reaches nominal significance.

## 5. Conclusions

This study is the first to describe the relationship between the Pomak population dietary habits and cardiovascular risk factors. The subpopulation represented in this study is an isolated population with many traditions and customs preserved during their sociocultural evolution, driven by a strong adherence to the Muslim religion. The “high in sugars” and “balanced” characteristic dietary patterns, identified in our study, are associated with cardiovascular risk factors such as blood pressure, glucose, and cholesterol levels. Although the compounds of these patterns, such as red meat and sweets, have aggravating effects on cardiovascular risk, they play a central role in the social life of the community as part of several religious ceremonies. Thus, the guidelines to decrease the incidence of cardiovascular risk should be adjusted and applied with respect to the traditions of this population, perhaps by providing healthier alternatives and improving effective policy making. 

## Figures and Tables

**Table 1:** Anthropometric, clinical, and dietary characteristics of the study population by sex.

	Total		Men		Women		*p*
	*n*	Mean (sd)	*n*	Mean (sd)	*n*	Mean (sd)	
%	1702		522	(30.7)	1180	(69.3)	
Age (years)	1664	44.87 (15.48)	519	48.71 (15.89)	1145	43.13 (14.97)	<0.001
Current weight (kg)	1638	72.8 (14.35)	510	78.63 (13.42)	1128	70.16 (13.98)	<0.001
Standing height (cm)	1635	162.97 (9.62)	508	172.62 (7.89)	1127	158.61 (6.73)	<0.001
Waist circumference (cm)	1588	92.17 (15.1)	496	96.14 (12.35)	1092	90.37 (15.87)	<0.001
WHR	1586	0.87 (0.1)	494	0.94 (0.08)	1092	0.84 (0.1)	<0.001
BMI (kg/m^2^)	1632	27.52 (5.42)	508	26.37 (4.14)	1124	28.03 (5.83)	<0.001
BMI status	1632		508	(31.1)	1124	(68.9)	
Underweight	52	(3.2)	7	(1.4)	45	(4.0)	<0.001
Normal weight	486	(29.8)	185	(36.4)	301	(26.8)	
Overweight	583	(35.7)	227	(44.7)	356	(31.7)	
Obese	511	(31.3)	89	(17.5)	422	(37.5)	
SBP (mmHg)	1265	131.21 (16.63)	433	133.27 (15.73)	832	130.14 (16.98)	0.001
DBP (mmHg)	1272	83.12 (11.01)	436	83.47 (10.38)	836	82.94 (11.32)	0.398
Food consumption (servings/week)							
Refined cereals	1607	29.6 (9.2)	500	30.2 (9.2)	1107	29.3 (9.2)	0.090
Full fat dairy	1625	18.2 (7.3)	504	18.8 (7.9)	1121	17.9 (7.0)	0.026
Vegetables	1624	16.5 (6.3)	504	16.8 (6.4)	1120	16.4 (6.3)	0.310
Sweets	1610	14.2 (6.2)	497	14.7 (5.8)	1113	14.0 (6.4)	0.031
Coffee	1626	10.7 (4.9)	504	11.5 (4.3)	1122	10.3 (5.1)	<0.001
Fresh fruits	1626	9.5 (8.5)	504	9.4 (8.6)	1122	9.5 (8.5)	0.784
Red meat	1624	7.5 (3.6)	503	7.7 (4.0)	1121	7.5 (3.3)	0.190
Fish	1621	2.7 (1.8)	499	2.7 (1.8)	1122	2.7 (1.8)	0.456
Potatoes	1626	2.6 (1.5)	504	2.7 (1.6)	1122	2.6 (1.5)	0.292
Legumes	1626	1.8 (1.1)	504	1.8 (1.1)	1122	1.8 (1.1)	0.975

**Table 2:** The most prevalent diseases by sex.

Disease *N* (%)	Total 1702	Men 522 (30.7)	Women 1180 (69.3)	*p*
Hypertension	444 (26.1)	139 (26.6)	305 (25.8)	0.735
Hyperlipidemia	284 (16.7)	102 (19.5)	182 (15.4)	0.036
Hypothyroidism	147 (8.6)	11 (2.1)	136 (11.5)	<0.001
Diabetes mellitus II	125 (7.3)	53 (10.2)	72 (6.1)	0.003
Coronary heart disease (CHD)	115 (6.8)	61 (11.7)	54 (4.6)	<0.001
Heart failure	112 (6.6)	41 (7.9)	71 (6.0)	0.159
Arthritis	127 (7.5)	39 (7.5)	88 (7.5)	0.992
Presbyopia	131 (7.7)	58 (11.1)	73 (6.2)	<0.001
Migraines	129 (7.6)	25 (4.8)	104 (8.8)	0.004
Mental Health Issues				
Depression	177 (10.4)	39 (7.5)	138 (11.7)	0.008

**Table 3:** Sociodemographic and lifestyle characteristics by sex.

	Total		Men		Women		*p*
	*n*	(%)	*n*	(%)	*n*	(%)	
Marital status	1629		506	(31.1)	1123	(68.9)	
Married	1413	(86.7)	439	(86.8)	974	(86.7)	<0.001
Single	153	(9.4)	61	(12.1)	92	(8.2)	
Widowed	63	(3.9)	6	(1.2)	57	(5.1)	
Educational attainment	1625		499	(30.7)	1126	(69.3)	
No education	178	(10.9)	29	(5.8)	149	(13.2)	<0.001
Primary	1174	(72.2)	348	(69.6)	826	(73.3)	
Secondary	237	(14.6)	100	(20.0)	137	(12.2)	
Tertiary	36	(2.2)	22	(4.4)	14	(1.2)	
Total years of education *, mean (SD)	967	6.86 (2.42)	303	7.45 (2.99)	664	6.59 (2.06)	<0.001
Smoking habits	1621		506	(31.2)	1115	(68.8)	
Current smokers	257	(15.9)	232	(45.8)	25	(2.2)	<0.001
Former smokers	103	(6.4)	98	(19.4)	5	(0.5)	
Never smokers	1261	(77.8)	176	(34.8)	1085	(97.3)	
		**Mean (SD)**		**Mean (SD)**		**Mean (SD)**	
Smoking starting age	340	16.72 (3.79)	315	16.61 (3.72)	25	18.08 (4.45)	0.062
Quit smoking age	109	41.5 (12.44)	105	41.87 (12.25)	4	32 (15.6)	0.120
Total smoking years	304	25 (14.07)	280	25.8 (14.02)	24	15.67 (11.25)	0.001
Pack-years *	274	30.5 (24.7)	251	31.9 (24.8)	23	15.5 (18.3)	<0.001
PAL *	1600	1.06 (0.13)	499	1.12 (0.19)	1101	1.03 (0.09)	<0.001
ee	1626	1864.4 (467.4)	502	2120.1 (523.6)	1124	1750.2 (389.3)	<0.001
EI/EE	1570	1.24 (0.44)	482	1.09 (0.37)	1088	1.31 (0.45)	<0.001
ei	1607	2199.2 (618.05)	495	2215.59 (649.31)	1112	2191.91 (603.78)	0.479

* Mann–Whitney test.

**Table 4:** Factor loadings derived from principal component analysis conducted with dietary variables (*N* = 1513).

	Principal Components			
Predicting variables	1	2	3	4
Fresh fruits	0.768	−0.074	0.192	−0.143
Sweets with fat	0.722	0.027	−0.065	−0.059
Beverages and juices	0.694	0.147	−0.047	−0.154
Starchy sweets	0.589	0.165	0.037	0.142
Processed meat	0.049	0.739	0.002	0.002
Ready pies	−0.065	0.678	−0.048	0.123
Refined breads	0.167	0.678	0.041	0.391
Fried potatoes	0.190	0.490	0.128	−0.278
Pasta and rice	−0.052	−0.106	0.716	0.133
Vegetables	0.206	0.024	0.639	0.247
Legumes	−0.405	0.073	0.571	0.014
Red meat	0.350	0.278	0.533	−0.192
Homemade pies	−0.277	−0.030	0.219	0.706
Full-fat cheese	0.155	0.493	0.042	0.597
Potatoes	−0.041	0.057	0.056	0.569
Variance explained (%)	16.3	14.0	11.0	10.7

**Table 5:** Biochemical analyses by dietary pattern.

				Pattern Adherence																			
				High in Sugar Diet					Quick Choices Diet					Balanced Diet					Homemade Pattern				
	Total			Low		High		P	Low		High		P	Low		High		P	Low		High		P
	n	Mean	(sd)	Mean	(sd)	Mean	(sd)		Mean	(sd)	Mean	(sd)		Mean	(sd)	Mean	(sd)		Mean	(sd)	Mean	(sd)	
Glucose (mg/dl)	1646	93.6	20.5	90.1	21.2	97.2	19.3	**<0.001**	94.5	21.1	92.8	19.9	0.129	92.3	20.0	95.1	21.0	**0.013**	93.7	20.1	93.6	21.0	0942
HDL Cholesterol (mg/dl)	1670	45.2	11.8	46.6	12.3	43.7	11.2	**<0.001**	45.3	11.9	45.0	11.7	0.584	45.4	11.6	44.9	12.0	0.492	44.7	11.7	45.6	11.9	0.197
LDL Cholesterol (mg/dl)	1663	118.5	34.2	116.6	34.8	120.2	33.3	0.054	120.4	35.0	116.3	32.9	**0.032**	118.5	33.6	118.3	34.5	0.901	117.7	34.0	119.1	34.2	0.453
Triglycerides (mg/dl)	1652	137.5	68.9	142.9	72.1	131.7	63.9	**0.003**	137.2	66.2	137.2	70.4	0.998	136.5	69.6	137.9	66.9	0.713	137.4	70.7	137.0	65.6	0.909
Insulin (uIU/ml)	1629	13.1	9.7	13.0	10.1	13.1	9.2	0.824	12.8	9.8	13.3	9.5	0.416	12.6	9.1	13.5	10.1	0.098	13.1	9.4	13.1	9.8	0.962
Total Cholesterol (mg/dl)	1668	192.5	39.0	193.1	38.9	191.7	38.1	0.517	194.6	39.4	190.1	37.4	0.032	192.0	37.9	192.8	39.1	0.728	191.6	39.0	193.3	38.0	0.432

Low versus high as defined by the median of the corresponding pattern score; P: *p*-value, HDL: High density lipoprotein, LDL: Low density lipoprotein, In bold: *p* -value < 0.05.

**Table 6:** Results from multiple linear regression analysis of data from POMAK-HELIC study that evaluated the association between dietary patterns and cardiometabolic indices (*N* = 1513).

	Model 1			Model 2			Adj-R sq	Model 3			Adj-R sq	Model 4			Adj-R sq
	β	CI	*p*	β	CI	*p*		β	CI	*p*		β	CI	*p*	
HDL (mg/dL)															0.16
High sugar	−1.39	−2.03,−0.75	**<0.001**	−1.03	−1.64,−0.42	**0.001**	0.15	−1.28	−2.02,−0.55	**0.001**	0.16	−1.18	−1.99,−0.36	**0.005**	
Quick	−0.43	−1.07,0.21	0.190	0.16	−0.46,0.78	0.604	0.15	0.23	−0.45,0.91	0.508	0.15	−0.02	−0.75,0.70	0.949	
Balanced	−0.23	−0.87,0.41	0.485	0.35	−0.26,0.95	0.262	0.15	0.40	−0.27,1.07	0.244	0.15	0.19	−0.52,0.90	0.597	
Homemade	0.35	−0.30,0.99	0.289	0.68	0.08,1.28	0.026	0.15	0.69	0.07,1.31	0.030	0.15	0.62	−0.01,1.25	0.054	
SBP (mmHg)															0.49
High sugar	2.87	1.84,3.89	**<0.001**	3.63	2.85,4.41	**<0.001**	0.47	3.36	2.42,4.30	**<0.001**	0.49	2.74	1.71,3.77	**<0.001**	
Quick	−1.10	−2.14,−0.06	0.037	−0.11	−0.93,0.71	0.791	0.43	−1.02	−1.90,−0.14	0.023	0.46	−0.53	−1.45,0.39	0.260	
Balanced	0.74	−0.30,1.78	0.163	−1.00	−1.80,−0.20	0.014	0.43	−2.10	−2.96,−1.24	**<0.001**	0.47	−1.59	−2.50,−0.69	**0.001**	
Homemade	1.06	0.02,2.10	0.045	−0.02	−0.81,0.78	0.968	0.43	−0.31	−1.12,0.51	0.459	0.46	−0.26	−1.06,0.54	0.527	
DBP (mmHg)															0.28
High sugar	1.65	0.99,2.32	**<0.001**	1.94	1.35,2.54	**<0.001**	0.27	1.56	0.84,2.28	**<0.001**	0.28	1.46	0.67,2.26	**<0.001**	
Quick	0.20	−0.48,0.87	0.569	0.71	0.10,1.32	0.023	0.25	0.20	−0.47,0.86	0.562	0.26	0.46	−0.25,1.17	0.208	
Balanced	0.37	−0.30,1.05	0.278	−0.48	−1.08,0.11	0.112	0.24	−1.22	−1.87,−0.56	**<0.001**	0.27	−0.85	−1.54,−0.15	0.017	
Homemade	0.51	−0.17,1.18	0.141	−0.01	−0.61,0.58	0.965	0.24	−0.23	−0.84,0.38	0.463	0.26	−0.17	−0.79,0.45	0.587	
Cholesterol (mg/dL)															0.11
High sugar	0.06	−2.03,2.15	0.956	0.51	−1.56,2.57	0.629	0.09	0.76	−1.72,3.23	0.550	0.11	−0.43	−3.17,2.30	0.756	
Quick	−2.63	−4.71,−0.54	0.014	−1.29	−3.37,0.79	0.224	0.09	−1.65	−3.91,0.62	0.153	0.11	−2.05	−4.49,0.39	0.100	
Balanced	0.31	−1.78,2.40	0.771	−1.41	−3.44,0.62	0.173	0.09	−1.46	−3.71,0.78	0.201	0.11	−1.88	−4.27,0.52	0.124	
Homemade	0.61	−1.48,2.71	0.565	−0.41	−2.42,1.61	0.691	0.09	−0.14	−2.22,1.95	0.897	0.11	−1.40	−2.52,1.71	0.710	
LDL (mg/dL)															0.11
High sugar	2.10	0.25,3.95	0.026	2.21	0.37,4.06	0.019	0.07	2.70	0.50,4.90	0.016	0.10	1.45	−0.97,3.89	0.240	
Quick	−1.92	−3.78,−0.07	0.042	−1.47	−3.34,0.40	0.122	0.07	−2.10	−4.12,−0.09	0.041	0.10	−2.04	−4.20,0.13	0.065	
Balanced	−0.37	−2.22,1.48	0.695	−1.80	−3.61,0.02	0.053	0.07	−2.16	−4.16,−0.17	0.033	0.10	−2.15	−4.28,−0.03	0.047	
Homemade	0.46	−1.39,2.31	0.627	−0.36	−2.17,1.44	0.693	0.07	−0.16	−2.02,1.70	0.169	0.09	−0.31	−2.18,1.57	0.748	
Glucose															0.30
High sugar	3.11	2.00,4.22	**<0.001**	3.37	2.30,4.43	**<0.001**	0.17	3.38	2.20,4.57	**<0.001**	0.29	3.44	2.14,4.75	**<0.001**	
Quick	−1.09	−2.21,0.04	0.058	−0.72	−1.81,0.36	0.192	0.14	−1.35	−2.44,−0.26	0.015	0.28	−0.35	−1.51,0.81	0.557	
Balanced	2.17	1.06,3.29	**<0.001**	0.98	−0.08,2.04	0.070	0.14	0.36	−0.72,1.45	0.511	0.28	0.99	−0.15,2.13	0.090	
Homemade	−0.06	−1.18,1.07	0.922	−0.80	−1.85,0.25	0.134	0.14	−1.25	−2.26,−0.24	0.015	0.28	−0.95	−1.95,0.06	0.066	
Insulin (μIU/mL)															0.05
High sugar	0.11	−0.42,0.64	0.675	−0.19	−0.73,0.34	0.479	0.05	−0.19	−0.84,0.46	0.566	0.05	0.09	−0.62,0.81	0.799	
Quick	0.58	0.05,1.11	0.032	0.55	0.01,1.09	0.044	0.05	0.68	0.09,1.27	0.025	0.05	0.73	0.09,1.37	0.025	
Balanced	0.29	−0.24,0.82	0.288	0.04	−0.48,0.57	0.877	0.05	0.12	−0.46,0.71	0.679	0.05	0.24	−0.39,0.86	0.461	
Homemade	−0.07	−0.60,0.46	0.790	−0.19	−0.71,0.33	0.473	0.05	−0.17	−0.71,0.38	0.550	0.05	−0.11	−0.66,0.44	0.697	

Model 1: unadjusted; Model 2: Adjusted for age, sex, BMI; Model 3: Adjusted for age, sex, BMI, medication (where applicable), smoking and energy intake to energy expenditure ratio; Model 4: Adjusted for age, sex, BMI, medication (where applicable), smoking, energy intake to energy expenditure ratio, the remaining food patterns scores; CI: confidence interval, P: *p*-value, Adj-R sq: adjusted R square, BMI: Body mass index, SBP: Systolic blood pressure, DBP: Diastolic blood pressure, HDL: High density lipoprotein, LDL: Low density lipoprotein; In bold: *p*-value < 0.0125.

**Table 7:** Logistic regression analysis results of the association of quartiles of dietary components with pathological groups of low HDL, SBP, DBP, LDL cholesterol, and glucose levels (*N* = 1513).

		MODEL 1		MODEL 2	
	1st	2nd	3rd	2nd	3rd
	Ref.	OR (95%CI)–P		OR (95%CI)–P	
	**High in Sugars Diet Score Tertiles**			
Low HDL	1.00	**1.51 (1.09,2.09)–0.013**	**2.00 (1.38,2.90)–<0.001**	**1.42 (1.01,2.00)–0.043**	**1.84 (1.24,2.75)–0.003**
SBP groups	1.00	**2.27 (1.50,3.44)–<0.001**	**2.87 (1.74,4.72)–<0.001**	**2.07 (1.32,3.23)–0.001**	**2.40 (1.38,4.17)–0.002**
DBP groups	1.00	**1.85 (1.25,2.73)–0.002**	**2.55 (1.60,4.07)–<0.001**	**1.87 (1.23,2.84)–0.003**	**2.61 (1.55,4.39)–<0.001**
LDL groups	1.00	1.19 (0.85,1.66)–0.321	**1.71 (1.14,2.55)–0.009**	1.06 (0.74,1.54)–0.757	1.47 (0.96,2.27)–0.079
Glucose groups	1.00	1.50 (1.00,2.25)–0.052	1.64 (1.03,2.62)–0.037	1.64 (1.06,2.55)–0.025	1.85 (1.11,3.08)–0.018
	**Balanced Diet Score Tertiles**				
SBP groups	1.00	**0.57 (0.38,0.85)–0.006**	**0.63 (0.42,0.97)–0.035**	**0.56 (0.37,0.86)–0.007**	0.72 (0.46,1.13)–0.153
DBP groups	1.00	0.84 (0.57,1.22)–0.350	0.84 (0.56,1.25)–0.387	0.86 (0.58,1.27)–0.433	0.96 (0.63,1.48)–0.857
LDL groups	1.00	**0.82 (0.59,1.15)–0.259**	**0.62 (0.43,0.88)–0.008**	0.81 (0.58,1.15)–0.240	**0.62 (0.43,0.89)–0.010**

P: *p*-value; OR: odds ratio, CI: confidence interval; In bold: *p*-value < 0.013; Ref.: reference category for both models; MODEL1 adjusted for age, sex, BMI, physical activity level, smoking, and energy intake; MODEL2 adjusted for age, sex, BMI, physical activity level, smoking, energy intake, and the remaining food components; HDL: high-density lipoprotein—low: <40 mg/dL; SBP: systolic blood pressure—threshold: 140 mmHG; DBP: diastolic blood pressure—threshold: 90 mmHG; LDL: low-density lipoprotein—threshold: 100 mg/dL; glucose threshold: 110 mg/dL.
